# Classification performances of two diode arrays for patient‐specific quality assurance of stereotactic body radiation therapy treatments based on absolute dose measurements in phantom

**DOI:** 10.1002/acm2.70167

**Published:** 2025-07-25

**Authors:** Stefania Linsalata, Jake H. Pensavalle, Franco Perrone, Patrizio Barca, Fabio Di Martino, Fabiola Paiar, Antonio C. Traino

**Affiliations:** ^1^ U.O. Fisica Sanitaria Azienda Ospedaliero Universitaria Pisana Pisa Italy; ^2^ Scuola di Specializzazione in Fisica Medica Università degli Studi di Pisa Pisa Italy; ^3^ U.O. Radioterapia Oncologica Università degli Studi di Pisa Pisa Italy

**Keywords:** ArcCHECK, PSQA, ROC, SBRT, SRS MapCHECK

## Abstract

**Background:**

Despite the rapid growth in the clinical practice, no specific recommendations on pre‐treatment patient‐specific quality assurance of volumetric modulated arc therapy‐based stereotactic body radiation therapy plans have been established.

**Purpose:**

In this contest, the study aims to identify optimal gamma analysis criteria and thresholds for the Sun Nuclear ArcCHECK and SRS MapCHECK arrays.

**Methods:**

Twenty SBRT plans were delivered on both devices per plan and field‐by‐field. The measurements were compared with calculations and Gamma Passing Rates (GPRs), obtained using global normalization in absolute dose and 10% threshold, with six different Dose Difference (DD) / Distance To Agreement (DTA) criteria stricter than those universally suggested by the Report of the AAPM Task Group No. 218, were recorded. Receiver Operating Characteristics analysis was performed on GPRs while varying the threshold from 0% to 100%, the agreement between calculations and absolute dose measurements, obtained with a IBA Razor chamber at the isocenter in phantom at different levels (i.e., 1%, 2%, 3%, 4%, and 5%), being the Ground Truth. Significance of the resulting Areas Under Curve (AUCs) against the random guess was tested.

**Results:**

AUCs obtained with ArcCHECK are generally more significant than with SRS MapCHECK, while those measured field‐by‐field are more significant than per plan. Within the considered DD/DTA criteria, the most discriminative ones are device‐specific, that is, 2%/2 mm or 1%/2 mm for ArcCHECK and 2%/1 mm or 1%/1 mm for SRS MapCHECK.

**Conclusions:**

Our results on ArcCHECK confirm the indication of the AAPM Task Group No. 218, while for SRS MapCHECK, acceptable discriminating capabilities are possible with DTA = 1 mm, suggesting that devices with native higher spatial resolution, preferred in SBRT for the better sampling of the dose distribution, require tighter DTA.

## 1 INTRODUCION

The employment of hypofractionated and extremely hypofractionated radiation therapy, referred as Stereotactic Ablative Body Radiosurgery (SABR) or Stereotactic Body Radiation Therapy (SBRT), has significantly grown in clinical practice during the last 20 years due to technological improvements and the increasing clinical experience on disease and side effects control.^1^

The majority of SBRT treatments are currently performed with C‐arm linacs, using Intensity Modulated Radiation Therapy (IMRT) or Volumetric Modulated Arc Therapy (VMAT), and draw from these techniques the same Patient Specific Quality Assurance (PSQA) methodologies, even though SBRT irradiation conditions are much more demanding than those in conventional fractionated IMRT/VMAT plans due to the higher doses and dose gradients and smaller fields involved. Consequently, Miften et al.,^2^ in the Report of the AAPM Task Group No. 218, suggested tighter tolerances for gamma analysis^3^ in pre‐treatment PSQA of SBRT plans than those for conventional fractionated IMRT/VMAT plans, even without any value specification.

Despite the numerous studies focused on pre‐treatment PSQA in SBRT, encompassing different topics such as device comparisons,^4^, ^5^, ^6^ assessment of specific devices,^7^, ^8^, ^9^ gamma analysis^10^, ^11^ or sensitivity to errors in delivery,^12^, ^13^, ^14^ no specific guidelines and recommendations have been already proposed as recently reported by Malatesta et al.^15^ The parameters used in gamma analysis^2^, ^3^ for a pre‐treatment PSQA of SBRT plan, such as the Dose Difference (DD), the Distance To Agreement (DTA) or the Gamma Passing Rates (GPRs) thresholds, are frequently derived empirically from the everyday experience of clinical medical physicists or, more correctly, using methods from statistical process control.^2^, ^10^, ^16^

A pre‐treatment PSQA is fundamentally performed to answer the question if a plan is dosimetrically acceptable, that is, if it possible to reproduce with sufficient accuracy the calculated dose distribution. In gamma analysis,^2^, ^3^ setting up a GPR threshold, such that dose distribution comparisons exploiting GPRs above/under threshold classify the plans with a pass/fail status, is like performing a diagnostic test.

Classification performance of diagnostic tests are usually assessed trough the Receiver Operating Characteristics (ROC) analysis^17^ that permits to combine sensitivity (i.e., the capability of the test to correctly identify a disease when it is really present) and specificity (i.e., the capability of the test to rule out a disease when it is really absent), the Area Under the ROC Curve (AUC) being used to measure the diagnostic accuracy of the test.

Among the studies that have applied ROC analysis to GPRs in the attempt to explore sensitivity of the device or determine unbiased threshold criteria for gamma analysis, most of them examined GPRs of PSQA for intentionally modified plans.^14^, ^18^, ^19^, ^20^ Carlone et al.^18^ performed ROC analysis of GPRs derived from PSQA of IMRT prostate plans with MapCHECK, introducing Multi Leaf Collimator (MLC) positioning random errors within ±0.5, ±1, ±2, and ±3 mm SD. They reported that gamma analysis detects MLC misalignments >3 mm and suggest optimal thresholds for the five considered gamma criteria as unbiased. Analogously, Tattenberg et al.^14^ considered ROC curves from GPRs obtained with ArcCHECK for different amounts of MLC leaf position, gantry angle, collimator angle, jaw position, dose output, and Dosimetric Leaf Gap (DLG) random errors. They found that error detectability, based on AUCs, was good or excellent (AUC > 0.8) for DLG errors greater than ±0.4 mm, for MLC position errors greater than 1 mm and gantry angle errors greater than 0.6 deg, while the detectability fails (0.5 ≤ AUC < 0.6) for all the considered amount of errors of the collimator angle (within ±1.5 deg), the jaw position (within ±3.0 mm for upper and ±1.5 mm for lower jaw) and the dose output (within ±1.5%).

McKenzie et al.^21^ performed ROC analysis of GPRs form six different methods of PSQA in a population of 24 IMRT plans. The Ground Truth (GT) to assess their performance across all thresholds was based on multiple independent ion chamber measurements in phantom. They found that common clinical thresholds were not adequate to accurately identify dosimetrically unacceptable plans and, based on AUC measurements, some explored dosimetric methods for PSQA (namely, ion chamber, radiographic films, helical diode array and anterior‐delivered cumulative 2D diode array) performed better than anterior delivered field‐by‐field or planned gantry angle cumulative delivered on 2D diode array. Moreover, they determined optimal cutoffs for the different methods, pointing out their variability among devices.

With an approach conceptually similar to McKenzie et al.,^21^ our study aims to assess the classification performances of the gamma analysis performed with two commercial diode arrays widely used in pre‐treatment PSQA of SBRT plans, by identifying the optimal DD/DTA criteria and GPR thresholds, assuming the absolute dose agreement between measurements and Treatment Planning System (TPS) calculations at the isocenter in phantom to be the GT for ROC analysis.

The study encompasses GPRs derived, cumulatively, that is, per plan, and field‐by‐field, with six different combination of DD and DTA criteria tighter or equal to those suggested as universal in the AAPM Task Group No. 218 for gamma analysis of conventional fractionated IMRT/VMAT plans (i.e., global normalization in absolute dose with DD criterion = 3%, DTA criterion = 2 mm and 10% dose threshold).

The aim is to contribute to the identification of universally recognized reference criteria for SBRT pre‐treatment PSQA.

## 2 METHODS

Twenty SBRT plans were considered for the study. All of them were VMAT treatments for a TrueBeam v. 2.7 (Varian; Palo Alto, CA), mounting a 120 High Definition (HD) MLC (Varian; Palo Alto, CA), with two to four arcs. The plans were calculated at 6 MV FFF with the AcurosXB algorithm (Varian; Palo Alto, CA) on the Eclipse v. 16.1 TPS (Varian, Palo Alto, CA) at resolution grid of 1 mm. For all the considered SBRT plans, treatment sites, number of arcs, prescription doses, and number of fractions are reported in Table 1, with some descriptive parameters suggested by the ICRU 91.^22^ All the plans had a single isocenter located in the middle of the GTV and were optimized to obtain uniform dose in the PTV or at least in the GTV when required by OAR spearing.^23^

**TABLE 1:** Description of the main characteristics of the SBRT plans.

Patient ID	PD (Gy)	NF	Tumor Site	NA (non‐copl)	PI (%)	D_98%_ (Gy)	D_50%_ (Gy)	D_2%_ (Gy)	V_PTV_ (cm^3^)	V_PIV_ (cm^3^)	V_PTV∩PIV_ (cm^3^)	HI	CI
1	25	5	Abdomen	2	98%	24.4	25.0	25.6	37.7	39.20	35.30	0.05	1.19
2	48	8	Thorax	2	99%	33.4	46.5	48.7	59.8	42.01	34.50	0.33	2.11
3	50	5	Thorax	2	97%	47.9	50.1	51.5	84.2	84.05	79.10	0.07	1.13
4	24	3	Thorax	2	96%	14.3	23.9	24.9	135.3	129.71	109.70	0.44	1.46
5	40	5	Thorax	2	97%	38.8	40.3	41.5	54.3	58.13	51.20	0.07	1.20
6	35	5	Abdomen	2	98%	34.2	35.0	35.7	44.0	43.85	39.00	0.04	1.27
7	30	5	Abdomen	2	98%	29.4	30.0	30.7	62.1	62.10	57.40	0.04	1.17
8	30	3	Pelvis	2	98%	29.2	30.0	30.7	26.9	27.64	25.20	0.05	1.17
9	50	5	Thorax	2	97%	48.6	50.0	51.3	17.7	18.62	16.60	0.05	1.20
10	40	5	Thorax	2	97%	38.5	40.4	41.4	63.3	62.82	58.40	0.07	1.17
11	27	1	Head‐Neck	3 (2)	87%	27.8	26.7	31.1	2.8	5.35	2.80	0.12	1.91
12	27	1	Head‐Neck	4 (3)	96%	26.0	26.7	28.1	1.3	1.51	1.10	0.08	1.62
13	27	1	Head‐Neck	3 (2)	96%	24.5	27.8	28.2	0.5	0.40	0.30	0.13	2.22
14	40	5	Head‐Neck	2	98%	39.3	40.0	40.8	10.3	11.10	10.00	0.04	1.14
15	40	5	Head‐Neck	2	98%	39.5	40.0	40.8	7.3	7.16	6.80	0.03	1.13
16	25	5	Head‐Neck	2	97%	23.3	25.1	25.9	8.1	7.86	7.10	0.10	1.26
17	20	5	Head‐Neck	2	95%	18.9	20.0	21.0	10.4	12.48	10.00	0.11	1.30
18	27	1	Head‐Neck	3 (1)	98%	24.5	26.9	27.6	2.3	1.45	1.30	0.11	1.97
19	45	3	Thorax	2	94%	43.7	45.6	47.6	7.0	9.05	7.00	0.09	1.29
20	50	5	Thorax	2	97%	48.4	50.0	51.4	11.2	12.20	10.30	0.06	1.29

Abbreviations: CI, conformity index; D_V%_ [Gy] (i.e., D_98%_, D_50%_, D_2%_), doses at specified percentage of the PTV volume; HI, homogeneity index; NA (non‐colp), number of Arcs per Plan with the number of non‐coplanar arcs in curved brackets; NF, number of fractions; PD, prescribed dose; PI, prescription isodose referred to the plan maximal dose; V_PIV_, volume encompassed by the prescribed isodose; V_PTV∩PIV_, volume of the intersection between PTV and PIV; V_PTV_, volume of the PTV.

It is worth noting that none of the considered plans was effectively delivered to patients, and, since all the considered plans are VMAT, in the following the terms “field” and “arc” have the same meaning.

A pre‐treatment PSQA of all the considered plans was repeatedly acquired with two commercial diode arrays, namely, ArcCHECK (Sun Nuclear; Melbourne, FL, USA) and SRS MapCHECK (Sun Nuclear; Melbourne, FL, USA),^24^ in different ways:

1. By delivering all plan fields on the ArcCHECK device, housing its PMMA MultiPlug accessory (Sun Nuclear; Melbourne, FL). This condition is referred as ArcCHECK Cumulative (ACC).
2. By delivering single plan fields on the ArcCHECK device, housing its PMMA MultiPlug accessory. This condition is referred as ArcCHECK Field‐by‐Field (ACF).
3. By delivering all plan fields on the SRS MapCHECK device, parallel to the treatment couch, in its StereoPHAN PMMA accessory (Sun Nuclear; Melbourne, FL). This condition is referred as SRS MapCHECK Cumulative (SMCC).
4. By delivering single plan fields on the SRS MapCHECK device, parallel to the treatment couch, in its StereoPHAN PMMA accessory. This condition is referred as SRS MapCHECK Field‐by‐Field (SMCF).

The ArcCHECK and the SRS MapCHECK were previously calibrated to ensure a uniform response of the diodes at 6 MV, following the instructions of the manufacturer. Dose calibration was obtained by irradiating both arrays to a known dose at 6 MV FFF.

The LINAC output was measured before every measurement session and was within ±0.5% of the reference values.

Because non‐coplanar field measurements in ArcCHECK are discouraged due to the risk of the device electronics irradiation, while they are possible in SRS MapCHECK, all arcs were measured without couch rotation to avoid different irradiation geometries for different devices.

In other different sessions we measured, cumulatively and field‐by‐field, for the same set of plans (without couch rotation), the absorbed dose‐to‐water at the isocenter in the MultiPlug PMMA accessory within the ArcCHECK, following the methodology described by Seuntjens et al.^25^ and suggested also in the IAEA Report TRS No. 483.^26^ For this purpose, we used a PTW Unidos electrometer (PTW The Dosimetry Company; Freiburg, Germany)^27^ and a IBA Razor Chamber (IBA Dosimetry; Schwarzenbruck, Germany),^28^ calibrated in absorbed dose to water at the IBA standard laboratory and characterized by active volume of 0.01 cc, cavity length of 3.6 mm and cavity radius of 1 mm.

Since in our TPS it is not possible to outline a Region of Interest (ROI) sufficiently small to reproduce the exact volume of the microchamber, with the aim of optimizing the results and avoiding volume effects, the measured doses have been compared with calculated punctual doses at different cubic voxels of side 1, 1.5, 2, 2.15 and 2.5 mm (i.e., the TPS calculation grid), 2.15 mm corresponding to the cubic square of the active volume of the chamber.

The calculated point dose values at the isocenter obtained with the 2 mm grid (i.e., the maximum recommended grid size by the AAPM TG101^29^) have been successively used for comparison with the ionization chamber point measurements.

The Patient Software v 8.4 (Sun Nuclear; Melbourne, FL) was used to perform gamma analysis between measured and calculated dose distributions in phantoms at 1 mm grid for all the considered experimental conditions (i.e., ACC, ACF, SMCC, SMCF). GPRs, obtained using global normalization in absolute dose and a 10 % dose threshold, with six different DD/DTA criteria equal to or tighter than those suggested as universal by the AAPM Task Group No. 218^2^ namely, 3%/2 mm, 2%/2 mm, 1%/2 mm, 3%/1 mm, 2%/1 mm, 1%/1 mm, were recorded.

ROC analysis was performed on GPRs plotting in the *x*‐axis “1‐specificity” and in the *y*‐axis the “sensitivity” while varying the GPR threshold from 0% to 100%. In this specific case, the “sensitivity” (i.e., the “true positive fraction”) is the ratio between all the plans/arcs characterized by a GPR below the threshold and those which fail the dose agreement at the isocenter in phantom. Conversely, the “specificity” is the ratio between all the plans/arcs characterized by a GPR above the threshold and those which pass the dose agreement at the isocenter in phantom, while “1‐specificity” represents the “false positive fraction”, that is, the ratio between all the plans/arcs characterized by a GPR below the threshold and those which pass the dose agreement at the isocenter in phantom.

The agreement between calculations and absolute dose measurements at the isocenter in phantom is the GT for the ROC analysis, the underlying idea being that a dosimetrically acceptable plan should have at least good agreement in absolute dose at the isocenter. Different levels of agreement have been analyzed, namely, 1%, 2%, 3%, 4%, and 5%.

For each of them and the four experimental conditions (i.e., ACC, ACF, SMCC, SMCF), six different ROC curves (i.e., each for the six considered DD/DTA criteria combinations) have been calculated for a total of 120. AUC and Optimal Threshold (OPTh), obtained by minimizing the distance between the ROC curve and the upper left corner of the graph, have been estimated for each curve, while the significance of each AUC against the null hypothesis of AUC = 0.5 (random guess) was estimated by a Mann–Whitney *U*‐test.

To investigate the feasibility of correlating measurements from both instruments while maintaining optimal discrimination capabilities, potential relationships between GPRs obtained for the considered plans/fields with various criteria have been explored through Spearman permutation correlation analysis,^30^, ^31^ that is, a robust non‐parametric method used to assess the statistical significance (*p*_perm_ <0.05) of the Spearman rank correlation coefficient (*r*_S_), particularly effective when dealing with small sample sizes and based on random shuffle of ranks to build the null hypothesis population (*H*_0_: *r*_S _= 0) for testing the actual observed ranks against.

Because the chamber response is field size dependent for small fields,^26^, ^32^ all the considered plans were analyzed in terms of two complexity metrics^33^ related to the field aperture, namely, the Mean Equivalent Field Size (MEFS) and the Mean Square Root Area (MSRA).

For each single control point (CP), the Equivalent Field Size (EFS_CP_) is related to the single CP area (A_CP_) and perimeter (P_CP_) as follows:

(1) $$EFS_{CP}=4A_{CP}/P_{CP}$$

with

(2) $$A_{CP}=\sum_{\left(Leaf\;Pair=1\right)}^{M}Width_{Leaf\;Pair}PairOpening_{Leaf\;Pair}$$

(3) $$\begin{array}{cc} P_{CP} & =\sum_{\left(Leaf\#=1\right)}^{N}2\;Width_{Leaf}+|LeafOpening_{\left(Leaf\#\right)}\\ & \;-LeafOpening_{\left(Leaf\#-1\right)}| \end{array}$$

Being *M* and *N*, respectively, the number of leaf pairs and the number of leaves, opened in each CP.

MEFS and MSRA are thus defined as follows:

(4) $$MEFS=\frac{1}{CP_{tot}}\sum_{CP=1}^{CP=CP_{tot}}\;EFS_{CP}$$

(5) $$MSRA=\frac{1}{CP_{tot}}\sum_{CP=1}^{CP=CP_{tot}}\sqrt{A_{CP}}$$

CP_tot_ being the total number of CP in the plan (for cumulative analysis) or in the arc (for field‐by‐field analysis). For each considered plan/arc, a homemade Python script was used to calculate both MEFS and MSRA.

Finally, correlations between these field aperture‐related metrics and absolute dose agreement in phantom were studied.

## 3 RESULTS

**FIGURE 1:**
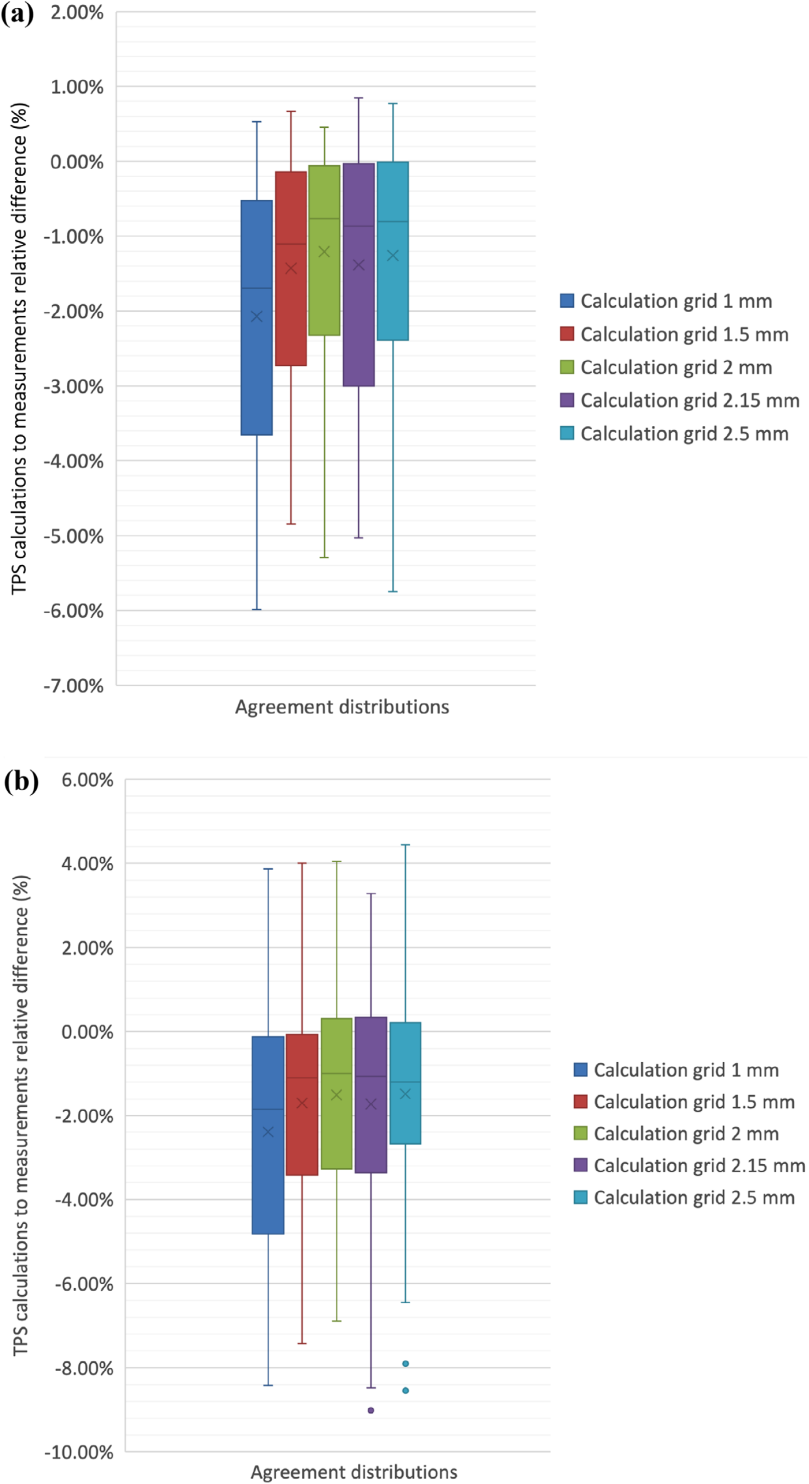
Agreements between calculated and measured absorbed dose‐to‐water at the isocenter in phantom. Box and whiskers plots reporting the distribution, over the 20 considered plans, of the agreements between calculated isocenter doses, at different TPS grids, namely, 1.0, 1.5, 2.0, 2.15 and 2.5 mm, and absolute dose‐to‐water measurements with the IBA Razor ionization chamber in the center of the Sun Nuclear MultiPlug PMMA insert of the ArcCHECK array. (a) The agreements for cumulative measurements, (b) agreements for measurements per field.

In Figure 1, the calculated point doses with different grids (i.e., 1.0, 1.5, 2.0, 2.15, and 2.5 mm) were compared to the measurements obtained with the IBA Razor ionization chamber in the center of the Sun Nuclear MultiPlug PMMA insert of the ArcCHECK array cumulatively (Figure 1a) and field‐by‐field, (Figure 1b).

Small differences appear in the dose agreements with varying the grid size, however, larger differences are observed for the smallest grid of 1 mm and the field‐by‐field condition (Figure 1b). As consequence, for the subsequent analyses, based on the agreement between TPS calculations and the microchamber measurements, the 2 mm calculation grid, which is still recommended for calculation of SBRT plans^29^ and corresponds to a sample volume of 0.008 cc, was chosen as a compromise between optimal agreement and closeness to the micro‐chamber volume of 0.01 cc.

**FIGURE 2:**
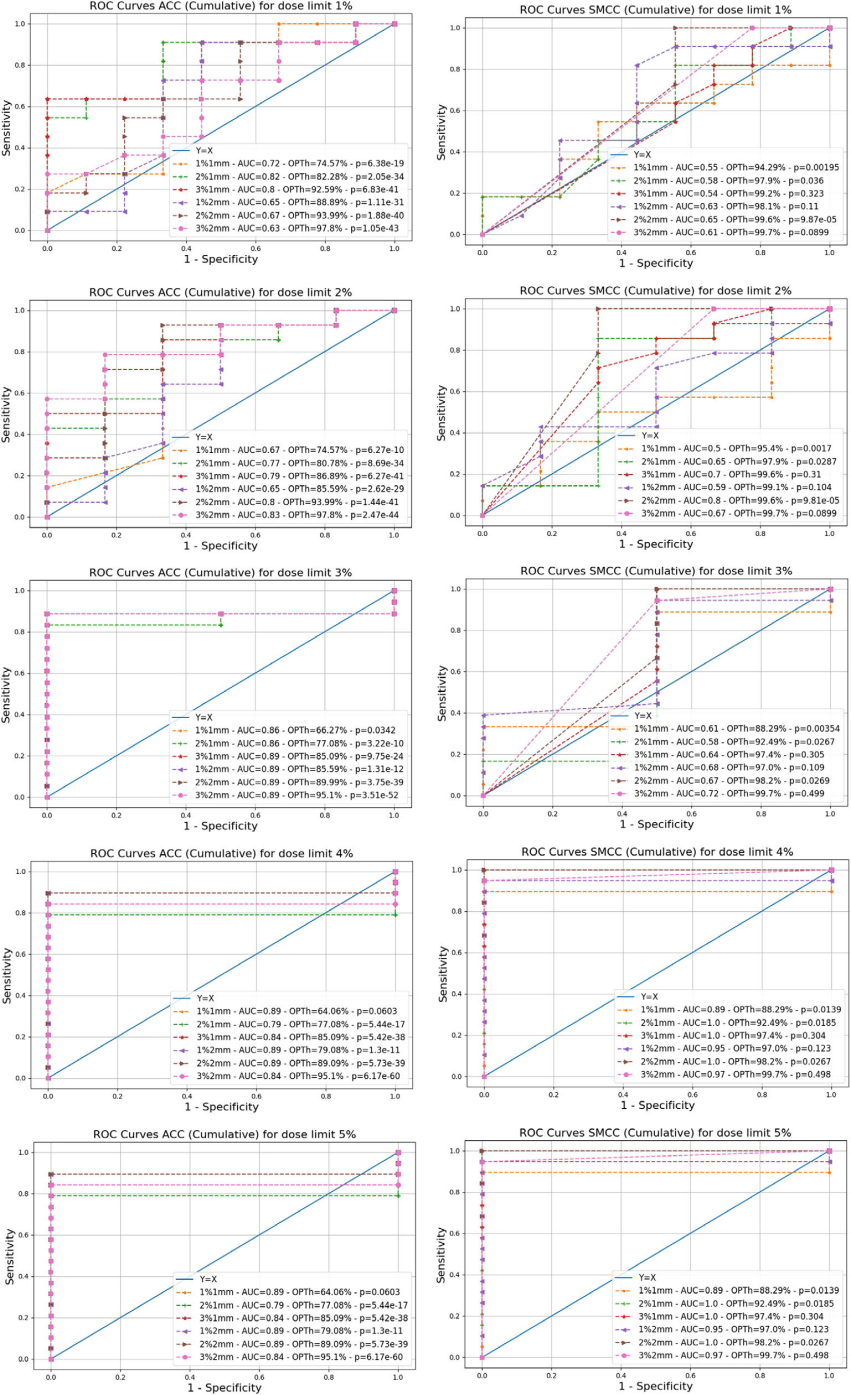
ROC curves for cumulative irradiation conditions. This figure reports all the ROC curves obtained for the considered cumulative irradiation conditions, namely, ACC, on the left, and SMCC, on the right. The ROC curves have been calculated considering as GT different levels of agreement between the doses estimated by the TPS at the isocenter in phantom (calculation grid 2 mm) and the IBA Razor microchamber measurements in the MultiPlug accessory of the ArcCHECK. Curves for different GT levels, namely, 1%, 2%, 3%, 4%, and 5%, are reported in rows. Each graph contains six ROC curves for the six considered DD/DTA gamma analysis criteria for the ACC (left) and the SMCC (right) measurement conditions. Within each graph, for each ROC curve, we have reported the corresponding AUC and OPTh. Abbreviations: ACC, ArcCHECK Cumulative; AUC, area under the curve; DD, dose difference; DTA, distance to agreement; GT, ground truth; OPTh, optimal threshold; ROC, receiver operating characteristics; SMCC, SRS MapCHECK Cumulative; TPS, treatment planning system.

**FIGURE 3:**
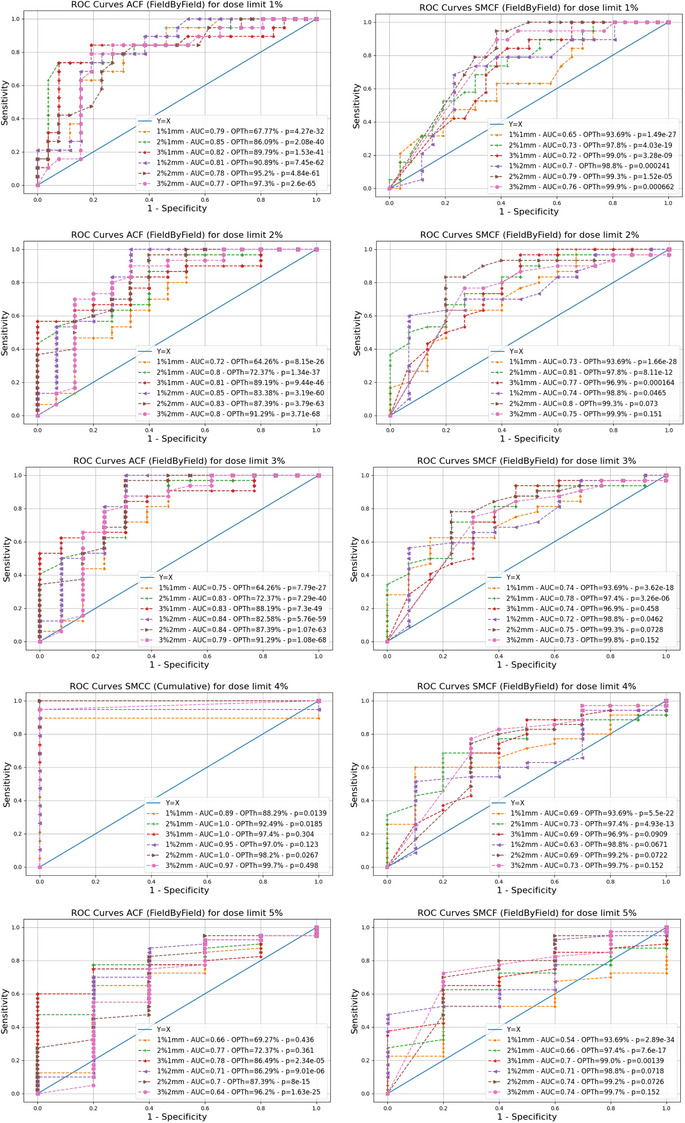
ROC curves for field‐by‐field irradiation conditions. This figure reports all the ROC curves obtained for the considered per field irradiation conditions, namely, ACF, on the left, and SMCF, on the right. The ROC curves have been calculated considering as GT different levels of agreement between the doses estimated by the TPS at the isocenter in phantom (calculation grid 2 mm) and the IBA Razor microchamber measurements in the MultiPlug accessory of the ArcCHECK. Curves for different GT levels, namely, 1%, 2%, 3%, 4%, and 5%, are reported in rows. Each graph contains six ROC curves for the six considered DD/DTA gamma analysis criteria for the ACF (left) and the SMCF (right) measurement conditions. Within each graph, for each ROC curve, we have reported the corresponding AUC and OPTh. Abbreviations: ACF, ArcCHECK Field‐by‐Field; AUC, area under the curve; DD, dose difference; DTA, distance to agreement; GT, ground truth; OPTh, optimal threshold; ROC, receiver operating characteristics; SMCF, SRS MapCHECKField‐by‐Field; TPS, treatment planning system.

Figure 2 presents the ROC analyses for the five GT criteria considered and the experimental conditions ACC (left) and SMCC (right). Similarly, Figure 3 reports the results obtained for the conditions ACF (left) and SMCF (right). For each curve, the AUC and the OPTh are displayed. Each AUC's significance against the random guess (AUC = 0.5) has been tested, with results shown in Table 2.

**TABLE 2:** Probability of the Mann–Whitney *U*‐test for AUCs against the random guess (AUC = 0.5).

Probability ACC	GT = 1%	GT = 2%	GT = 3%	GT = 4%	GT = 5%
**1%/1** **mm**	1.28E‐18	1.25E‐09	6.84E‐02	1.21E‐01	1.21E‐01
**2%/1** **mm**	4.09E‐34	1.74E‐33	6.43E‐10	1.09E‐16	1.09E‐16
**3%/1** **mm**	1.37E‐40	1.25E‐40	1.95E‐23	1.08E‐37	1.08E‐37
**1%/2** **mm**	2.22E‐31	5.23E‐29	2.63E‐12	2.60E‐11	2.60E‐11
**2%/2** **mm**	3.76E‐40	2.88E‐41	7.51E‐39	1.15E‐38	1.15E‐38
**3%/2** **mm**	2.10E‐43	4.93E‐44	7.02E‐52	1.23E‐59	1.23E‐59

Probability SMCC	GT = 1%	GT = 2%	GT = 3%	GT = 4%	GT = 5%
**1%/1** **mm**	3.91E‐03	3.40E‐03	7.08E‐03	2.79E‐02	2.79E‐02
**2%/1** **mm**	7.19E‐02	5.74E‐02	5.33E‐02	3.71E‐02	3.71E‐02
**3%/1** **mm**	6.45E‐01	6.20E‐01	6.10E‐01	6.08E‐01	6.08E‐01
**1%/2** **mm**	2.20E‐01	2.09E‐01	2.17E‐01	2.46E‐01	2.46E‐01
**2%/2** **mm**	1.97E‐04	1.96E‐04	5.39E‐02	5.34E‐02	5.34E‐02
**3%/2** **mm**	1.80E‐01	1.80E‐01	9.98E‐01	9.97E‐01	9.97E‐01

Probability ACF	GT = 1%	GT = 2%	GT = 3%	GT = 4%	GT = 5%
**1%/1** **mm**	8.55E‐32	1,63E‐25	1.56E‐26	1.39E‐30	8.73E‐01
**2%/1** **mm**	4.16E‐40	2,69E‐37	1.46E‐39	9.43E‐45	7.22E‐01
**3%/1** **mm**	3.06E‐41	1,89E‐45	1.46E‐48	5.11E‐53	4.68E‐05
**1%/2** **mm**	1.49E‐61	6,39E‐60	1.15E‐58	9.95E‐61	1.80E‐05
**2%/2** **mm**	9.68E‐61	7,59E‐63	2.14E‐63	8.15E‐62	1.60E‐14
**3%/2** **mm**	5.20E‐65	7,42E‐68	2.17E‐68	1.17E‐69	3.26E‐25

Probability SMCF	GT = 1%	GT = 2%	GT = 3%	GT = 4%	GT = 5%
**1%/1** **mm**	2.97E‐27	3.31E‐28	7.25E‐18	1.10E‐21	5.77E‐34
**2%/1** **mm**	8.06E‐19	1.62E‐11	6.52E‐06	9.87E‐13	1.52E‐16
**3%/1** **mm**	6.56E‐09	3.28E‐04	9.16E‐01	1.82E‐01	2.78E‐03
**1%/2** **mm**	4.82E‐04	9.29E‐02	9.25E‐02	1.34E‐01	1.44E‐01
**2%/2** **mm**	3.04E‐05	1.46E‐01	1.46E‐01	1.44E‐01	1.45E‐01
**3%/2** **mm**	1.32E‐03	3.02E‐01	3.04E‐01	3.04E‐01	3.03E‐01

Abbreviations: ACC, ArcCHECK Cumulative irradiation condition; ACF, ArcCHECK Field‐by‐field irradiation condition; AUC, area under curve; SMCC, SRS MapCHECK Cumulative irradiation condition; SMCF, SRS MapCHECK Field‐by‐field irradiation condition.

Generally, AUCs obtained with ArcCHECK are more significant than those obtained with SRS MapCHECK, while AUCs measured field‐by‐field are more significant than for composite measurements. Specifically, the percentage of significant AUCs (*p*‐value < 0.05) against the random guess was 90% for ACC, 33% for SMCC, 93% for ACF, and 53% for SMCF.

The configurations yielding the highest significant AUCs over the different considered GTs, corresponding to the most discriminative DD/DTA criteria, are:

1. For ACC
  - within all GTs, 1%/2 mm and 2%/2 mm at 4%, both with an AUC of 0.895 and OPTh of 79.1% and 89.1% respectively.
  - restricted to GT = 3%, 1%/2 mm and 2%/2 mm, both with an AUC of 0.889 and OPTh of 85.6% and 90.0% respectively.
2. For SMCC
  - within all GTs, 2%/1 mm at both 4% and 5%, with an AUC of 1.00 and OPTh of 92.5%.
  - restricted to GT = 3%, 1%/1 mm, with an AUC of 0.611 and OPTh of 88.3%.
3. For ACF
  - within all GTs, 1%/2 mm at 4%, with an AUC of 0.891 and OPTh of 82.5%.
  - restricted to GT = 3%, 1%/2 mm, with an AUC of 0.844 and OPTh of 82.6%.
4. For SMCF
  - within all GTs, 2%/1 mm at 2%, with an AUC of 0.808 and OPTh of 97.8%.
  - restricted to GT = 3%, 2%/1 mm, with an AUC of 0.780 and OPTh of 97.4%.

It is noteworthy that these derived optimal DD/DTA criteria have for ArcCHECK DTA = 2 mm, whereas for SRS MapCHECK DTA = 1 mm.

Additionally, the highest AUCs do not necessarily correspond to configurations with the lowest *p*‐values (see Figure 2, Figure 3, and Table 2). This distinction underscores our choice of prioritizing the value of the AUC, for those AUCs with *p*‐values < 0.05, to optimize the discriminatory power of the ROC analysis, rather than solely minimizing *p*‐values.

**FIGURE 4:**
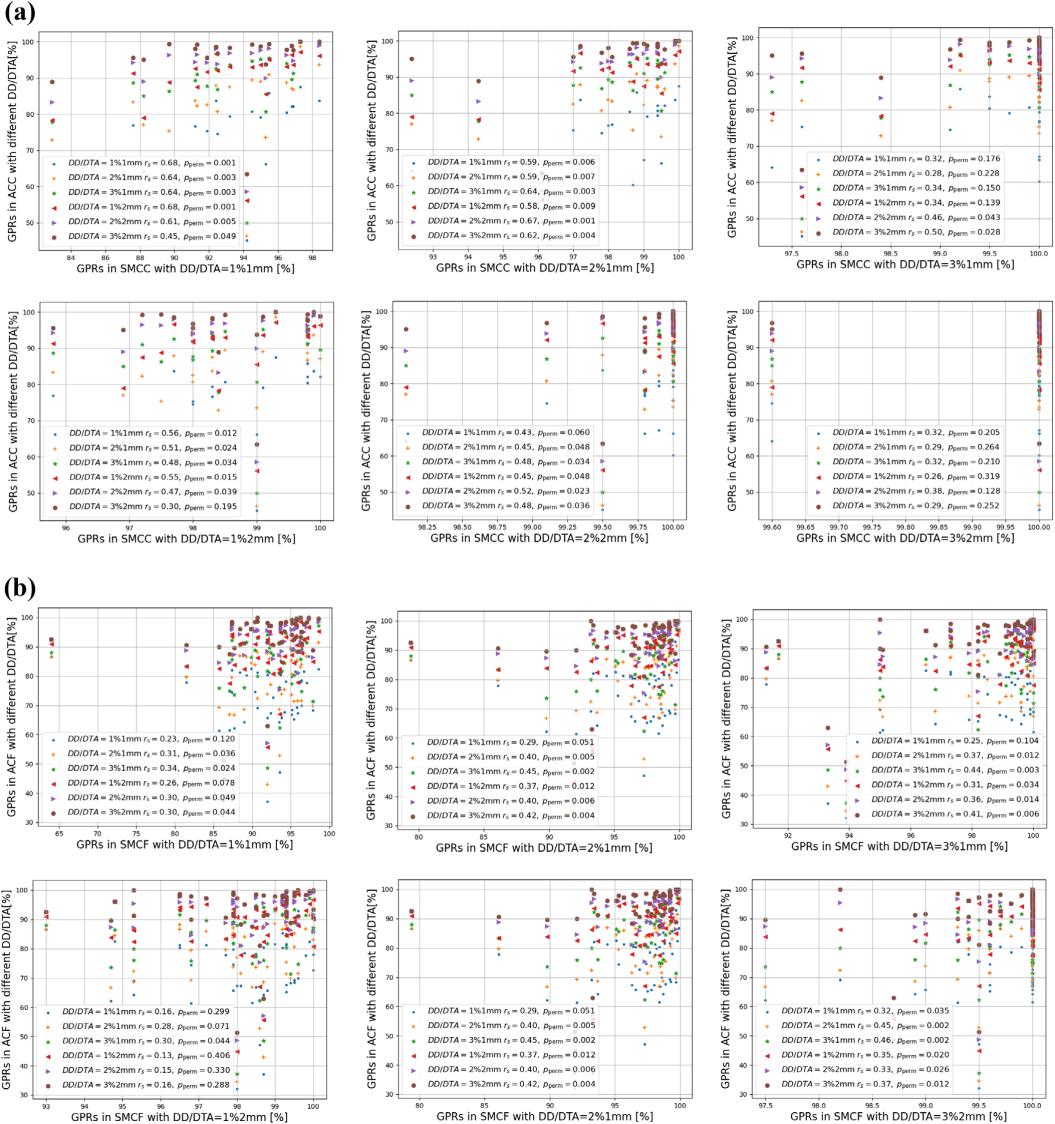
Correlations between GPRs: ArcCHECK against SRS MapCHECK. This figure shows, in the upper part (a), six graphs with the scatter plots reporting, for the 20 considered plans, the correspondence of GPRs registered with all the considered DD/DTA criteria combination in the ACC condition (*y*‐axis) over a single DD/DTA criteria combination in the SMCC condition (*x*‐axis). Different graphs correspond to the six considered DD/DTA criteria combinations in SMCC. Analogously, in part (b), six graphs report, for the 46 fields of the 20 considered plans, the correspondence of GPRs registered with all the considered DD/DTA criteria combination in the ACF condition (*y*‐axis) over a single DD/DTA criteria combination in the SMCF condition (*x*‐axis). Different graphs correspond to the six considered DD/DTA criteria combinations in SMCF. Correlations between GPRs obtained with various criteria for ArcCHECK and SRS MapCHECK, per plan and per field, based on the Spearman permutation testing, were investigated, reporting on the graphs, for each correlation, the value of the Spearman coefficient (*r*_S_) and the probability (*p*_perm_) of the Spearman permutation test.^30^, ^31^ Abbreviations: ACF, ArcCHECK Field‐by‐Field; ACC, ArcCHECK Cumulative; DD, dose difference; DTA, distance to agreement; GPRs, gamma passing rates; *p*_perm_, permutation test probability; *r*_S_, Spearman correlation coefficient; SMCC, SRS MapCHECK Cumulative; SMCF, SRS MapCHECK Field‐by‐Field; TPS, treatment planning system.

Correlation between GPRs obtained with various criteria for ArcCHECK against SRS MapCHECK, per plan and field, is generally significant based on the Spearman permutation testing (*p*_perm_‐values < 0.05) except for the loosest criteria of 3%/2 mm on SRS MapCHECK in cumulative analyses (see Figure 4a).

**FIGURE 5:**
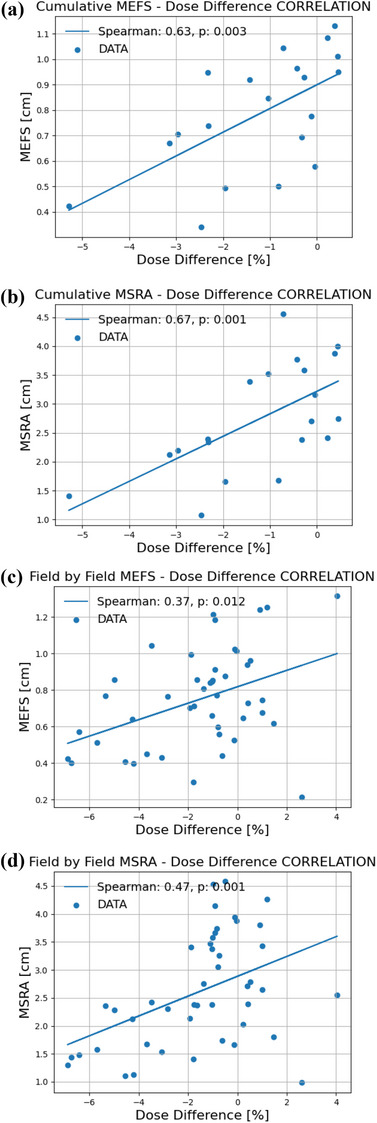
Relationships between dose agreement and aperture‐related metrics. This figure reports the correlation analysis, based on the Spearman correlation coefficient between dose agreement, that is, TPS calculations versus measurements with the IBA Razor chamber, and the aperture related metrics, namely, the MEFS defined in Equation (4) and the MSRA defined in Equation (5). Correlations are reported for cumulative analysis in (a) and (b), and for field‐by‐field analysis in (c) and (d). Abbreviations: MEFS, mean equivalent field size; MSRA, mean square root area; TPS, treatment planning system.

To study the relationship between dosimetric agreement and field aperture metrics, correlations between field aperture complexity metrics (MEFS and MSRA) and absolute dose measurements were analyzed under cumulative and field‐by‐field conditions (Figure 5). The statistically significant positive slope of the linear regression curves indicates that the smaller values of these metrics are associated with worse dosimetric agreement.

## 4 DISCUSSION

The main purpose of this study is to evaluate, through ROC analysis, the classification performances of GPRs derived from gamma analysis with two different devices, namely the ArcCHECK and SRS MapCHECK, for PSQA in SBRT plans delivered on a C‐arm linac, against absolute dose measurements at the isocenter in a phantom.

While the majority of the studies apply ROC analysis to GPRs assess classification performance of PSQA for intentionally modified plans,^14^, ^18^, ^19^, ^20^ we preferred to analyze clinical plans without the introduction of intentional delivery errors. In fact, in our opinion, studying the performance of a particular device for PSQA by inducing delivery errors, often well outside the clinical machine tolerance table, makes little sense, being their real occurrence highly unlikely.

In this sense, the approach of our study is conceptually similar to that used by McKenzie et al.,^21^ the main difference being that we considered VMAT SBRT plans and two devices, ArcCHECK and SRS Map CHECK, the ArcCHECK being analyzed by the above‐mentioned authors.

Conversely to McKenzie et al.,^21^ which based their ROC analysis on multiple independent ion chamber measurements in phantom, we chose as GT the agreement between TPS calculations and measurements in a single measurement point. This choice is often obligatory for SBRT plans due to restricted irradiated volume, such as in our case (see Table 1), where it is justified by the homogeneous prescription to the GTV.

In fact, in our opinion, assuming that a nearly uniform dose prescription is assigned to the GTV^23^ and that the isocenter is within the GTV, an acceptable plan from a dosimetric perspective should at least exhibit dose agreement at the isocenter in the phantom. This dose agreement criterion has then been chosen as GT for ROC analysis. This request is in accordance with the general goal of the overall accuracy of a radiotherapy treatment,^34^ and it is particularly important for SBRT plans due to the high doses delivered per fraction.

On the other hand, measuring absolute dose for a clinical SBRT VMAT plan it is not trivial due to several issues, the most relevant ones being the small fields dosimetry,^26^, ^32^ which is still a stimulating challenge especially for MLC modulated fields and the absolute dose measurements in solid phantom needed for the verification of clinical plans. Good measurements of modulated small fields in plastic phantoms are also needed for the accurate commissioning of the TPS.

Figure 5 resumes the limitations of this study, meanwhile suggesting future work and challenges. The clear relationship between the absolute dose agreement and MEFS or MSRA could be caused by the lack of our MLC model in the TPS for very small modulated fields (see Table 1), dependence of the ionization chamber response to the effective field size, and bias on the absolute dose measurements in plastic phantom. All of them certainly contribute to the results and deserve to be considered separately. Despite the mentioned limitations, Figure 5 suggests MEFS and MSRA as possible metrics to account for complexity of SBRT plans.^16^, ^33^ Especially, MEFS is a good candidate for an attempt to extend to modulated small fields the current approach of static small fields dosimetry^26^, ^32^ and to introduce for modulated fields appropriate corrections of the detector response. In fact, the study of Das et al.^35^ recently demonstrated the equivalence between the classical equivalent field concept. 4A/P and $S\mathrm{clin}=\sqrt{\left(FWHM(x)\cdot FWHM(y\right)}$ ^26^ except for very small non‐squared fields (≤10 mm), the result being confirmed through Monte Carlo (MC) calculations by Ding and Das.^36^ These results open the perspective of deriving new $k_{Q_{clin},Q_{msr}}^{f_{clin},f_{msr}}$ factors in terms of equivalent square field, with the advantages of using direct geometry information of the field derived from digital readouts of the linac.

It can be argued that the IBA Razor chamber is not the best choice for small field dosimetry; however, for ionization chambers, there is experience of measuring absolute dose in plastic phantom.^25^ Further measurements with different detectors, such as micro‐diamond or plastic scintillators, and MC calculations could contribute to understand fluence perturbation and spectral response for the detectors to small, MLC modulated fields.

Despite the mentioned limitations in the estimation of the dose agreement at the isocenter in phantom, we consider our approach still valid for evaluating the discrimination performances of the ArcCHECK and SRS MapCHECK using this dose agreement as GT for the ROC analysis, being our goal the comparison between two detector arrays for repeated measurements. Biases in the absolute dose agreement estimation will reasonably act on the analysis relative to each detector in the same extent.

Our results show that within the considered DD/DTA criteria, the most discriminative ones are device‐specific, that is, 2%/2 mm or 1%/2 mm for ArcCHECK and 2%/1 mm or 1%/1 mm for SRS MapCHECK, suggesting that the native higher spatial resolution of the device is the main reason to choose a tighter DTA criterion. On the other hand, a more relaxed DTA criterion for the device with better native spatial resolution does not improve its discriminating capabilities. Moreover, the optimal thresholds are criteria and device‐specific.

Surprisingly, AUCs for ArcCHECK are generally higher than those with SRS MapCHECK. This is true despite the generally higher GPRs obtained with SRS MapCHECK (original data available upon request), as already reported.^4^, ^10^, ^37^

Results upon the SMCC conditions appear uncertain (see Table 2 with poor significance of AUCs) and suggest preferring using this instrument for field‐by‐field analysis, as already reported by Rose et al.^37^

Our ROC analyses on ArcCHECK somewhat confirm the indication of the AAPM Task Group No. 218^2^, showing for the ACC condition, GT = 3%, which is reasonable to guarantee the required overall accuracy of the radiation therapy treatment,^22^, ^35^ and the 3%/2 mm gamma criteria suggested therein, an OPTh = 95.1% very close to the universal tolerance limit reported.

This statement does not apply on the results obtained with SRS MapCHECK, the universal criteria and threshold suggested by the AAPM Task Group No. 218^2^ being too weak.

Our data show that the use of detector arrays with higher spatial resolution in SBRT PSQA, with the aim to increase the number of measured points, should be accompanied by specific DD/DTA criteria and thresholds. This is in line with the recommendations of Malatesta et al.^15^ that suggest tailoring DD/DTA criteria to the available instruments by using narrow DTA (e.g., 1 mm) for high resolution detectors and eventually compensate for the demanding goal with a greater DD.

Our results on the correlation between GPRs obtained with ArcCHECK and SRS MapCHECK, per plan and per field, do not completely agree with other studies. Berk et al. reported no correlation between GPRs obtained with SRS MapCHECK and Gafchromic EBT3 films against the TPS for three DD/DTA criteria combination, that is, 5%/1 mm, 4%/1 mm, and 3%/1 mm.^16^ This result could be due to our choice to explore all possible correlations between different DD/DTA criteria instead of limiting the analysis to the same criteria with different detectors.

## 5 CONCLUSIONS

This study has aimed to investigate, through ROC analysis, the classification performance and consequently the most discriminating DD/DTA criteria of gamma analysis of two commercial diode arrays for PSQA on SBRT plans, namely, SunNuclear ArcCHECK, and SunNuclear SRS MapCHECK. The GT has been chosen as the agreement in absolute dose measurements at the isocenter in phantom with respect to TPS calculations. For 3% of this agreement maximal significant AUCs against random guess are: for ACC 1%/2 mm and 2%/2 mm, both with an AUC of 0.889 and OPTh of 85.6% and 90.0%, respectively, for ACF 2%/2 mm, with an AUC of 0.844 and OPTh of 82.6%, for SMCC, 1%/1 mm, with an AUC of 0.611 and OPTh of 88.3% and for SMCF 2%/1 mm, with an AUC of 0.780 and OPTh of 97.4%.

For ArcCHECK, the proposed criteria for both the considered conditions, that is, cumulative and field‐by‐field, are in line with the indications of the AAPM Task Group No. 218^2^ which considered mainly detectors with similar spatial resolution. For SRS MapCHECK, acceptable discriminating capabilities are possible with DTA = 1 mm. Provided that detectors with higher spatial resolution are preferred for SBRT PSQA to permit optimal dose distribution sampling, our results confirm the need of specific recommendations on SBRT PSQA tailored with respect to detector spatial resolution.^15^

## AUTHOR CONTRIBUTIONS

Stefania Linsalata participated in design of the study, data collection and measurements, data analysis and interpretation, and wrote and reviewed the manuscript. Jake H. Pensavalle participated in data collection and data analysis, wrote the Python scripts, and reviewed the manuscript. Franco Perrone participated in data collection and measurements and reviewed the manuscript. Patrizio Barca participated in calibration measurements, data interpretation, and reviewed the manuscript. Fabio Di Martino contributed to data interpretation and reviewed the manuscript. Fabiola Paiar contributed to study design and reviewed the manuscript. Antonio C. Traino conceived the idea of the study, provided guidance during experiments, reviewed all steps, and reviewed the manuscript.

## CONFLICT OF INTEREST STATEMENT

The authors declare no conflicts of interest.
